# P-1912. Training for tomorrow: Why an Infectious Diseases rotation must be mandatory to Internal Medicine Residency training

**DOI:** 10.1093/ofid/ofaf695.2081

**Published:** 2026-01-11

**Authors:** Rhea Bohra, Rajaeswaran Chinnamuthu, George M Abraham

**Affiliations:** Saint Vincent Hospital, Worcester, MA; Saint Vincent Hospital, Worcester, MA; Saint Vincent Hospital, Worcester, MA

## Abstract

**Background:**

Infectious Diseases (ID) bridges all clinical disciplines, yet remains inconsistently incorporated into Internal Medicine (IM) residency programs, often limited to electives without a standardized curriculum. As antimicrobial resistance (AMR) rises, previously controlled infections resurge, and immunocompromised populations grow, the need for foundational ID training is paramount. This study evaluates competencies and perceived educational value among residents in a program with a required ID rotation.

**Fig 1:**
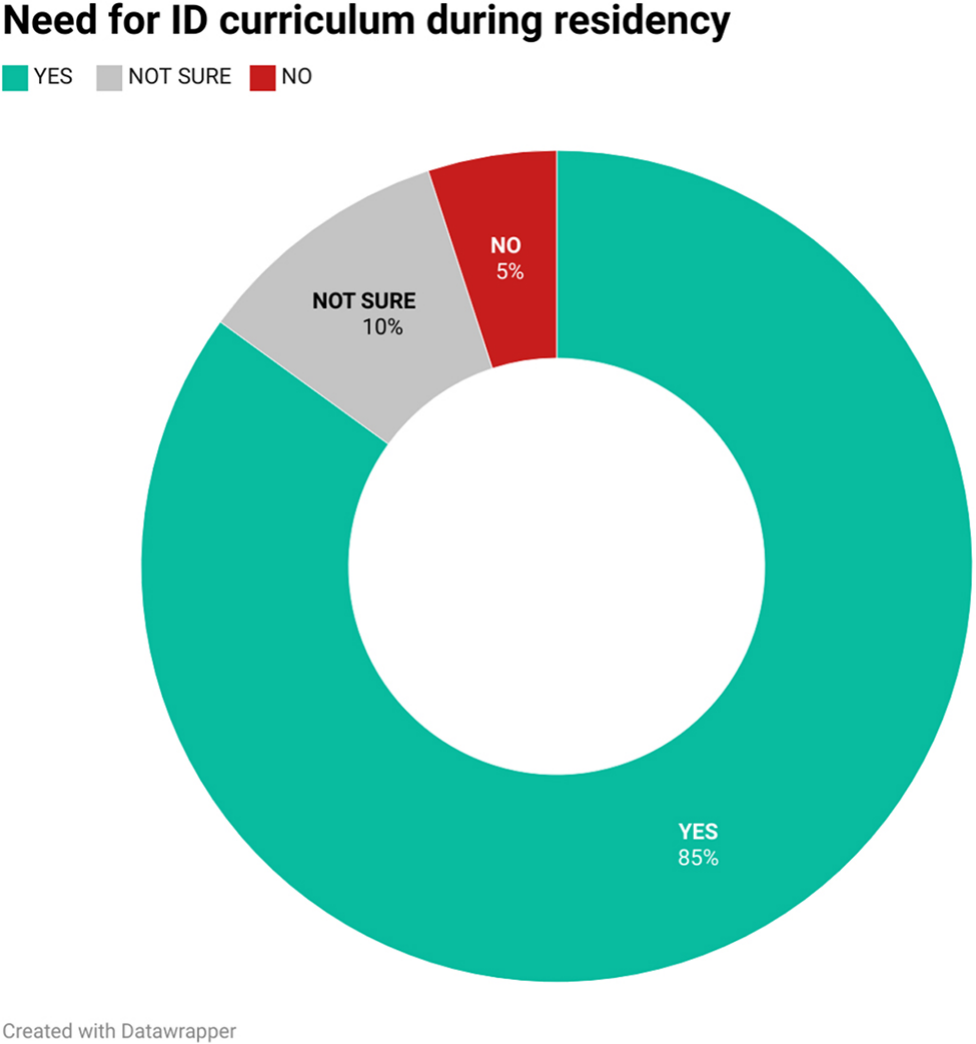
Piechart showing percentage of residents who note a robust ID curriculum will help future goals

**Fig 2:**
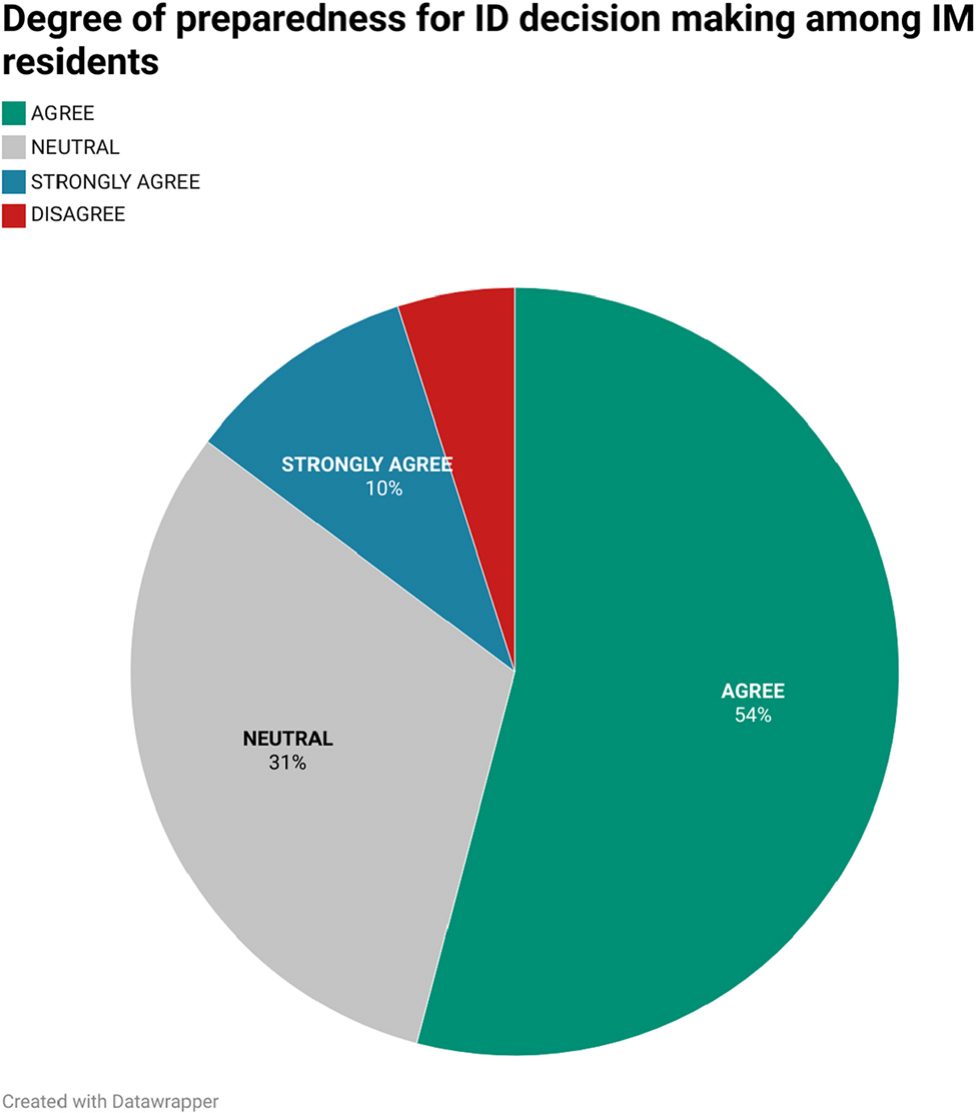
Piechart showing percentage of residents with degree of preparedness in ID clinical decision making

**Methods:**

An anonymous online survey was distributed to IM residents at Saint Vincent Hospital, covering demographics, self-assessed ID competencies, career goals, In Training Examination (ITE) impact, and feedback. Topics were based on a structured curriculum. Responses were rated on a 5-point Likert^®^ scale and analyzed in MS Excel^®^.

**Fig 3:**
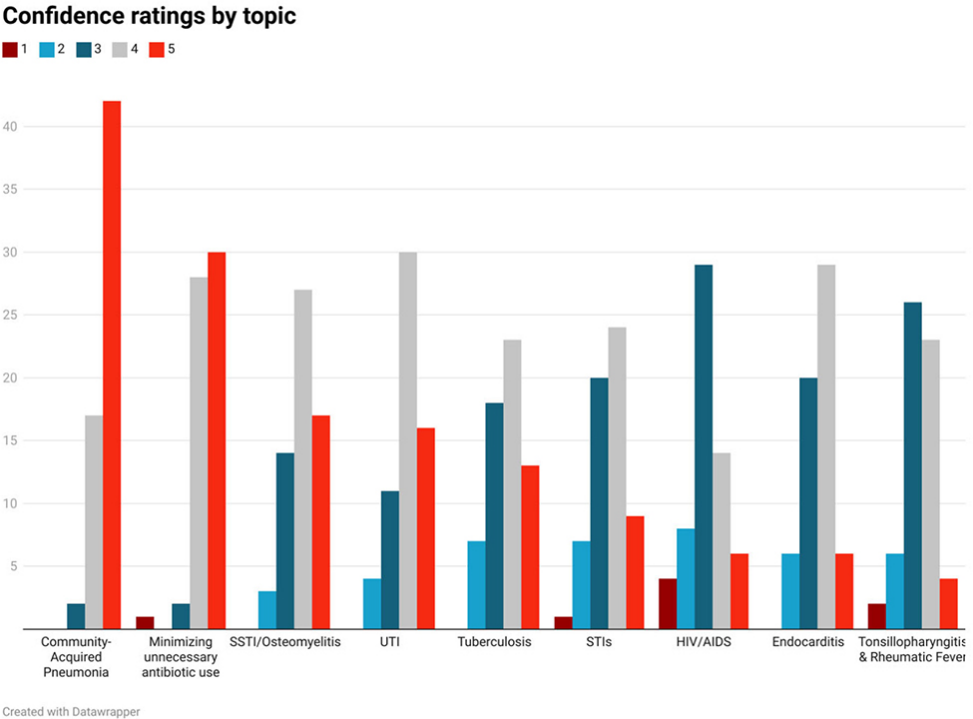
Bar chart showing degree of confidence among various ID topics of IM residents Degree of preparedness graded on a scale of 1-5 (1: Not confident, 5: Very Confident)

**Fig 4:**
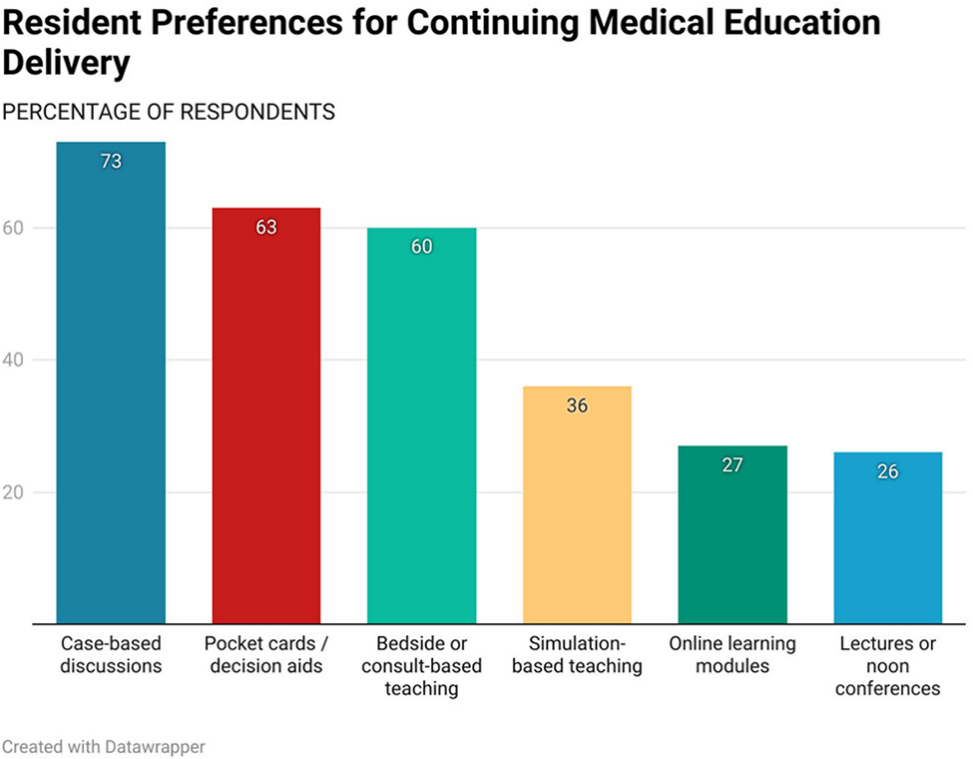
Bar chart showing preferences of educational tools for ID learning by IM residents

**Results:**

60 residents completed the survey; 73% had completed an ID rotation. Residents reported highest confidence managing community-acquired pneumonia and minimizing unnecessary antibiotic use, and lowest confidence in travel medicine, protozoal, and fungal infections. 64% felt well-prepared for ID decision-making, and 89% routinely reassessed antibiotics based on microbiologic data. 85% agreed a robust ID curriculum would support their careers. 55% had not completed an ID rotation before their ITE; those who had, noted improved performance. Residents identified a need for additional education in antimicrobial susceptibility interpretation, travel medicine, and HIV care, with case-based discussions and pocket aids preferred as learning tools.

**Conclusion:**

IM residents, regardless of career path, recognized the value of standardized, mandatory ID training for both clinical practice and academic development. Strengthening antimicrobial stewardship at the residency level is critical to reducing the AMR burden. Feedback from this survey will guide ongoing refinement of a dynamic, resident-centered curriculum. As resistant organisms rise and new pandemics loom, ID training can no longer remain elective. Embedding it as a core requirement is essential to developing physicians capable of delivering comprehensive care and advancing global health efforts.

**Disclosures:**

All Authors: No reported disclosures

